# Effect of COVID-19 pandemic on ART access and timely initiation in people living with HIV in 31 countries: a regression discontinuity design study

**DOI:** 10.1136/bmjopen-2025-112903

**Published:** 2026-03-10

**Authors:** Jihane Ben Farhat, Eugène Messou, Rohidas Borse, Diana Varela Bustillo, Metsekae Madimabe, Denis Nash, Helen Byakwaga, N Sarita Shah, Oliver Ezechi, Sanjay Pujari, Valdiléa G Veloso, Michael Hobbins, Gad Murenzi, Denna Mkwashapi, Brenna Hogan, Jun Yong Choi, Albert Minga, Brenda Crabtree-Ramírez, Christella Twizere, Lameck Diero, Jason Haw, Carolyn Bolton-Moore, Claudia P Cortes, Man-Po Lee, Safari Mbewe, Ellen Brazier, John M Humphrey, Keri N Althoff, Fabrice Bonnet, Diana Barger, Antoine Jaquet, IeDEA International epidemiology Databases to Evaluate AIDS

**Affiliations:** 1 Médecins Sans Frontières Department of Epidemiology and Training, Epicentre, Paris, France; 2 National Institute for Health and Medical Research (INSERM) UMR 1219, Research Institute for Sustainable Development (IRD) EMR 271, University of Bordeaux, Bordeaux, France; 3 Programme PACCI/ANRS Research Center, Abidjan, Côte d’Ivoire; 4 BJ Government Medical Center, Pune, India; 5 Hospital Escuela, Tegucigalpa, Honduras; 6 Instituto de Enfermedades Infecciosas y Parasitarias Antonio Vidal, Tegucigalpa, Honduras; 7 Desmond Tutu HIV Foundation, Mowbray, South Africa; 8 Institute for Implementation Science in Population Health, City University of New York, Graduate School of Public Health and Health Policy, New York, New York, USA; 9 University of Eldoret, Eldoret, Kenya; 10 Emory University, Atlanta, Georgia, USA; 11 Nigerian Institute of Medical Research, Lagos, Nigeria; 12 Institute of Infectious Diseases, Pune, India; 13 Instituto Nacional de Infectologia Evandro Chagas, Rio de Janeiro, Brazil; 14 SolidarMed, Lucerne, Switzerland; 15 School of Public Health, College of Medicine and Health Sciences, University of Rwanda, Kigali, Rwanda; 16 National Institute for Medical Research, Dar es Salaam, Tanzania, United Republic of; 17 Department of Epidemiology, Johns Hopkins Bloomberg School of Public Health, Baltimore, Maryland, USA; 18 Internal Medicine, Yonsei University College of Medicine, Seoul, Republic of Korea; 19 Centre médical de Suivi des Donneurs de Sang, Centre National de Transfusion Sanguine Côte d'Ivoire, Abidjan, Côte d\'Ivoire; 20 Departamento de Infectología, Instituto Nacional de Ciencias Médicas y Nutrición Salvador Zubirán, Mexico City, Mexico; 21 Centre National de Référence en matière de VIH/SIDA (CNR), Bujumbura, Burundi; 22 Immune Suppression Syndrome Clinic, Mbarara, Uganda; 23 Center for Infectious Disease Research, Lusaka, Zambia; 24 Infectious Disease, University of Alabama, Birmingham, Alabama, USA; 25 Fundación Arriarán, Santiago, Chile; 26 University of Santiago de Chile School of Medicine, Santiago, Chile; 27 Queen Elizabeth Hospital, Birmingham, UK; 28 Lighthouse Trust, Lilongwe, Malawi; 29 Department of Medicine, Indiana University, Indianapolis, Indiana, USA; 30 Service de Médecine Interne et Maladies Infectieuses, CHU de Bordeaux, Bordeaux, France; 31 Institut Pascal (UMR 6602), Université Clermont Auvergne, Centre National de la Recherche Scientifique, Clermont-Ferrand, France

**Keywords:** COVID-19, HIV & AIDS, Drug Therapy

## Abstract

**Abstract:**

**Objectives:**

The COVID-19 pandemic threatened global HIV Test and Treat Efforts. We assessed whether it affected (1) the number of antiretroviral therapy (ART) initiations and (2) the proportion of timely ART initiations in people living with HIV (PLWH) globally.

**Design:**

Quasi-experimental, regression discontinuity design using routinely collected data from HIV clinics.

**Setting:**

360 HIV care clinics across primary and secondary levels of care, participating in the International epidemiology Databases to Evaluate AIDS consortium, in 31 countries in Asia, Africa and the Americas.

**Participants:**

177 391 PLWH (≥18 years old) who initiated ART 2 years before and 1 year after the onset of the COVID-19 pandemic in their country.

**Primary and secondary outcome measures:**

The primary outcome was the number of ART initiations per week; the secondary outcome was the proportion of timely ART initiations (ie, ART initiated within 7 days of enrolment). We assessed changes in these outcomes in the 52 weeks after compared to the 104 weeks before the pandemic onset, defined using each country’s peak Oxford Stringency Index score between January and June 2020.

**Results:**

Among 177 391 newly enrolled PLWH, 129 743 initiated during the pre-pandemic and 47 648 post-pandemic onset. 72.5% of ART initiations were timely pre-pandemic whereas 82.3% were during the pandemic. Absolute number of ART initiations remained stable during the pandemic period in 25 of 31 countries but decreased significantly in six countries: India (−5.0 p, 95% CI −9.2 to −0.7), Rwanda (−10.0 p, −18.6 to −1.4), Malawi (−33.4 p, −54.1 to −12.3), South Africa (−130.8 p, −188.6 to −73.1), Zimbabwe (−12.9 p, −20.0 to −5.8) and Togo (−19.6 p, −39.1 to −0.1). The proportion of timely initiations was stable in all countries except in Kenya (+4.2 pp, 95% CI +0.3 to +8.1) and in Mozambique (+2.7 pp, +0.5 to +4.9), where it increased significantly.

**Conclusions:**

A deeper understanding of the factors that contributed to sustaining ART initiations, particularly in settings with stringent public health and social measures, is needed. These insights should inform preparedness strategies, resource allocation and policy development to ensure continuity of HIV services during future health emergencies, in line with World Health Organisation recommendations.

**STRENGTHS AND LIMITATIONS OF THIS STUDY**

- This large-scale, multicountry analysis relies on data from 31 countries across diverse regions, providing insight into how antiretroviral therapy (ART) initiation in people living with HIV was affected in both high-resource and low-resource settings at the onset of the COVID-19 pandemic.
- The use of the Oxford Stringency Index to define the onset of the pandemic allowed for a standardised measure of public health and social restrictions across countries and for the investigation of the hypothesis that public health responses may have indirectly affected delivery of non-COVID-19 services, such as HIV care.
- Our study used a Regression Discontinuity Design to estimate changes in ART initiations at the onset of the pandemic, strengthening causal inference by comparing ART initiation trends immediately before and after a clearly defined threshold.
- Our analysis did not account for pre-pandemic ART initiation trends, which may influence the interpretation of absolute differences across countries; moreover, countries with low weekly ART initiation counts may have had limited statistical power, reducing the precision of effect estimates.
- Our analysis pertains to new ART initiations, which do not encompass the full spectrum of HIV service delivery changes during the pandemic.

## Background

 The COVID-19 pandemic, which began in early 2020, has profoundly impacted societies and their populations. In an effort to reduce the spread of SARS-CoV-2, public health and social measures including various forms of social distancing—for example, lockdowns, shelter-in-place mandates (including severe movement restrictions), closures of public spaces, remote work, physical distancing, etc—were enacted on a global scale.^1^ In spite of these efforts, the rapid surge in cases of serious COVID-19 illness overwhelmed hospitals and intensive care units, leading to shortages of healthcare workers and resources. Additionally, disruptions in global supply chains contributed to delays in the availability of essential medicines, testing supplies and medical equipment.^2^ Non-essential surgeries and procedures, treatments and routine healthcare were cancelled or postponed to both accommodate acute COVID-19 care and protect others from infection. There was mounting evidence that the pandemic period, including related public health and social measures, had adversely affected the management of non-COVID-19 conditions such as cardiovascular disease, diabetes and cancer, threatening health outcomes in these patients.^3^,^5^

People living with HIV (PLWH), whether undiagnosed or diagnosed, were considered to be vulnerable during the COVID-19 pandemic due to HIV-related immune suppression, underlying disparities in risk factors, and/or potential interruptions in healthcare delivery, particularly access to antiretroviral therapy (ART).^6 7^ While initial reports from Europe and North America did not point to increased susceptibility or risk of severe COVID-19 in PLWH,^8^ subsequent studies found HIV status to be associated with poorer COVID-19-related outcomes (eg, severe disease, hospitalisation) and increased mortality.^9^,^13^ In 2021, The Global Fund to Fight AIDS, Tuberculosis and Malaria report highlighted substantial decreases in prevention services and HIV testing and raised concerns about access to ART during the pandemic.^14^ While the implementation of differentiated service delivery, extended visit intervals and telehealth helped maintain access to services for those in care in some^15 16^ but not all settings,^17 18^ substantial declines in ART initiations, often coupled with declines in HIV testing, were nevertheless reported.^18^,^21^ Early evidence on the COVID-19 pandemic’s impact on ART initiation/access was often self-reported, either from healthcare providers or PLWH/key populations or both, and/or fragmented, reflecting specific healthcare settings, cities, regions or countries. Furthermore, many studies conducted using existing observational databases (cohorts, electronic health records, claims) relied on year-over-year or retrospective comparisons and descriptive temporal approaches, which often fail to fully capture underlying trends and may be subject to temporal, selection and measurement biases.^22 23^

The Test and Treat Strategy, recommended by the World Health Organisation (WHO) since 2015,^24^ involves proactive HIV testing and immediate ART initation upon diagnosis, regardless of CD4 cell count or clinical stage. This approach aims to reduce morbidity, mortality and transmission by ensuring that all PLWH are linked to care as early as possible and receive effective ART. As the Test and Treat Strategy relies not only on the availability of health system resources (ie diagnostics, ART and trained personnel)^24^ but also their accessibility, including the ability of individuals to physically reach care, we hypothesised that the unprecedented circumstances of the COVID-19 pandemic may have disrupted both availability and accessibility. These disruptions could, in turn, have affected the delivery of non-COVID-19 services, including key aspects of HIV care such as ART initiations among those presenting for care and/or their timeliness. To investigate this hypothesis, we apply a regression discontinuity design (RDD) to assess the causal impact of the COVID-19 pandemic onset on the number and timeliness of ART initiations at clinics in 31 countries and interpret these results in light of the severity of public health and social measures implemented in each setting.

## Methods

### Study design and setting

**Figure 1:**
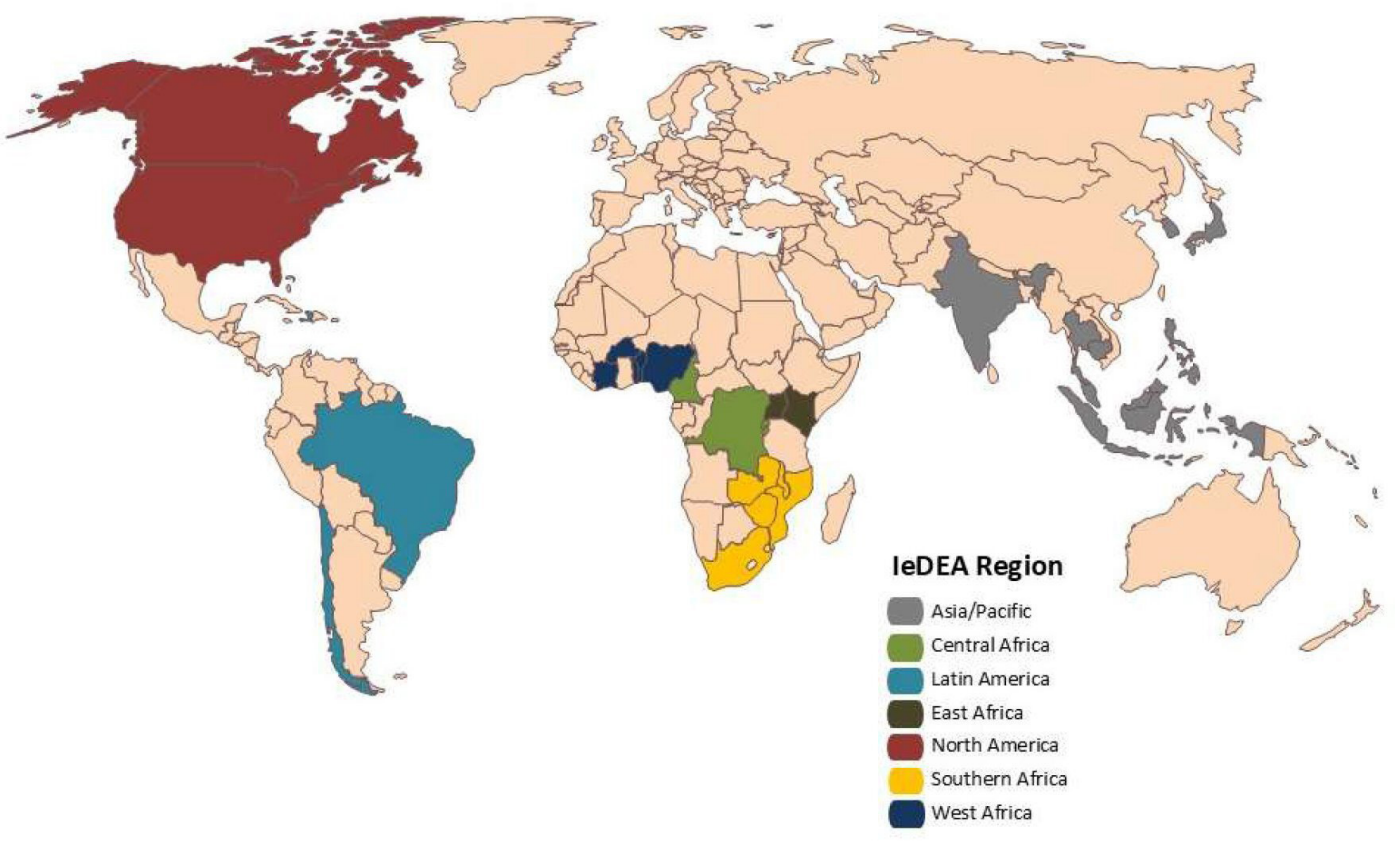
Countries included in the analysis by IeDEA region. IeDEA, International epidemiology Databases to Evaluate AIDS.

We conducted a retrospective analysis of anonymised, routinely collected, individual-level data within the International epidemiology Databases to Evaluate AIDS (IeDEA) collaboration. The IeDEA collaboration is an international research consortium that collects and harmonises patient-level data from HIV clinics and programmes in seven regions of the world (Asia-Pacific, Central Africa, East Africa, Southern Africa, West Africa, Latin America and North America) to better understand the epidemiology of HIV, treatment outcomes and the effects of interventions. We considered available data (all individuals living with HIV who presented for care at age 18 years or older) from 373 HIV clinics located in 35 countries. We excluded clinics with dates of database closure within 52 weeks of the pandemic onset (n=9) as well as those with insufficient observations (n=4 for the first outcome and then n=5 for the second outcome). A total of 360 clinics located in 31 countries in Asia, Africa and the Americas were ultimately included in the analyses (figure 1).

### Variables

#### Outcomes

The primary outcomes were the number of ART initiations and the proportion of timely ART initiations per week. The number of ART initiations was defined as the count of ART initiations among PLWH presenting for the first time at the clinic (enrolment), while timely ART initiation was defined as initiation within 7 days of presentation at the clinic.

### Pandemic period (exposure)

We used the Oxford Stringency Index to define the onset of the pandemic in each country. The Oxford Stringency Index Score is a composite measure that tracks the severity of government responses to COVID-19, including mobility restrictions, lockdowns, curfews, school closures, international travel bans and other public health and social measures. It ranges from 0 to 100, with higher values indicating greater levels of stringency. The pandemic onset was defined as the date corresponding to the peak Oxford Stringency Index Score, occurring between 1 January 2020 and 30 June 2020 (online supplemental annex 1), a period which generally reflected the first wave of the pandemic in the countries in question as well as the height of the public health response. Data were downloaded from the Oxford COVID-19 Government Response Tracker website to determine the peak Oxford Stringency Index score and corresponding date for each country.^25^ We used the date of clinic enrolment as a continuous eligibility assignment variable, classifying individuals enrolled before the country’s pandemic onset date as ‘unexposed’ and those enrolled on or after the onset date as ‘exposed’.

### Statistical analysis

We summarised participants’ demographic characteristics (age and sex) and calculated the number of ART initiations per week by period (before vs during the pandemic). We considered ART initiations to have been ‘timely’ if they occurred within 7 days of presentation at the clinic. We determined this interval by calculating the number of days between the date of presentation and the date of ART initiation. We then determined the proportion of timely initiated PLWH among the total number of ART initiations per week.

We employed an RDD to assess changes in both the average number of ART initiations (measured in absolute numbers) and the proportion of timely initiations (measured in percentage points) during the pandemic compared with the pre-pandemic period. This quasi-experimental pretest-posttest design seeks to determine the causal effects of an event or intervention (the COVID-19 pandemic) by choosing a threshold (eg, pandemic onset) and comparing observation before and after said threshold, controlling for temporal trends. RDD therefore provides an approximation of the counterfactual: What would have happened in the absence of COVID-19 related public health and social measures? ^26^

Our analysis considered a timeframe of 104 weeks before the pandemic and 52 weeks after its onset. To refine our estimates, we used local polynomial regression models based on data-driven bandwidth intervals calculated with the Imbens-Kalyanaraman (IK) method, using a rectangular (uniform) kernel for the estimation. The model was defined as follows:

E [Yi|Zi] = β0 + β1Zi + β2×1 [Zi≥0] + β3Zi×1 [Zi≥0]

where Yi is the probability of observing the outcome of interest, Zi is the number of days between the enrolment date and the start of the pandemic (negative for pre-pandemic period), and 1[Zi≥0] indicates outcome on or after the pandemic’s onset. Using the subset of observations within IK bandwidth intervals for each country, the effect of interest was the difference in local linear predictions at the threshold. We used the Rdrobust function to calculate conventional local-polynomial Regression Discontinuity effect point estimators and CIs.^27 28^ We visualised the estimated changes in number of ART/proportions of timely initiations during the COVID-19 pandemic period by country using dot-and-whisker plots. All statistical analyses were performed using STATA V.16.1 (StataCorp).

## Results

### Participants

During the study period, defined as 104 weeks before and 52 weeks after the pandemic onset (determined for each country), a total of 177 391 PLWH who presented for care at clinics were included in the analysis. Of whom, 129 743 initiated ART before the pandemic and 47 648 during the pandemic.

### Description of countries and dates of pandemic onset and levels of stringency

The following regions, countries and clinics were included in this analysis: Asia-Pacific (N=9 countries, n=16 clinics), Central Africa (N=4, n=21), Latin America (N=3, n=3), East Africa (N=2, n=59), Southern Africa (N=6, n=241), Northern America (N=2, n=13) and West Africa (N=5, n=7). Four countries (Taiwan, Argentina, Honduras and Mexico) were excluded from the analysis of the first outcome (number of ART initiations) due to an insufficient number of recorded ART initiations during the study period, resulting in an analytical sample of 31 countries. For the second outcome (timely ART initiation), five additional countries (Japan, Korea, Malaysia, Philippines and Brazil) were excluded due to insufficient weekly ART initiation data to reliably estimate proportions of timely initiation, leading to an analytical sample of 26 countries (online supplemental annex 2).

The exact dates reflecting the pandemic onset and the corresponding peak Oxford Stringency Index score for each country are presented in table 1. Across most regions, the peak stringency scores of the Oxford Stringency Index scores were observed between late March and mid-April 2020. However, a few exceptions should be noted: in Latin America, Brazil and Chile recorded their peak Oxford Stringency Index score in May 2020, and in Southern Africa, Mozambique’s peak score occurred in June 2020.

**Table 1:** Sex and median age of people living with HIV initiated on ART* (N=177 391), by period and region/country

	Characteristics						
				Female, n (%)		Median age (IQR)	
	Date of highest stringency score in the first 6 months of 2020	Peak stringency score in the first 6 months of 2020	Number of observations in the study period* N	Pre-COVID-19 period†	Pandemic period	Pre-COVID-19 period†	Pandemic period
Asia/Pacific							
Hong Kong	6 April 2020	66.67	356	42 (15.4)	16 (19.1)	38.4 (29.0–47.0)	40.6 (30.0–53.1)
Indonesia	24 April 2020	80.09	556	116 (28.8)	33 (21.6)	34.1 (28.2–42.1)	32.5 (27.4–39.6)
India	22 March 2020	100.00	1125	403 (42.9)	85 (45.7)	40.1 (32.5–48.3)	42.1 (33.8–49.8)
Japan	16 April 2020	47.22	278	5 (3.1)	4 (3.5)	34.8 (27.8–44.9)	35.4 (30.4–44.4)
Cambodia	10 April 2020	76.85	399	105 (35.5)	35 (34.0)	28.5 (21.9–39.4)	32.5 (25.8–41.7)
Korea	6 April 2020	82.41	119	–	2 (4.6)	31.2 (24.4–41.6)	31.4 (25.4–45.7)
Malaysia	27 March 2020	78.70	241	16 (10.0)	10 (12.4)	33.3 (26.8–42.0)	35.6 (31.3–43.5)
Philippines	22 March 2020	100.00	218	9 (4.4)	1 (8.3)	29.8 (25.3–34.3)	34.9 (29.3–38.4)
Thailand	3 April 2020	76.85	1088	181 (22.9)	61 (20.4)	33.2 (25.7–43.8)	32.4 (25.4–43.3)
Central Africa							
Burundi	22 March 2020	13.89	1156	480 (59.5)	203 (58.2)	35.7 (27.4–45.7)	32.9 (26.9–40.6)
Cameroon	18 April 2020	71.30	2298	890 (55.5)	371 (53.5)	36.9 (29.7–45.2)	37.8 (30.1–45.7)
Democratic Republic of Congo	6 April 2020	80.56	1299	525 (65.9)	348 (69.3)	42.4 (35.3–50.5)	42.7 (33.0–51.3)
Rwanda	21 March 2020	90.74	2679	1088 (57.8)	521 (65.5)	32.8 (26.6–39.9)	31.7 (26.1–38.5)
Latin America							
Brazil	5 May 2020	81.02	629	58 (12.7)	16 (9.4)	28.9 (23.7–38.1)	30.8 (24.4–38.3)
Chile	15 May 2020	81.02	893	60 (8.4)	20 (11.1)	30.2 (26.1–36.5)	35.9 (28.8–44.5)
Haiti	19 April 2020	93.52	2854	1270 (57.5)	354 (54.8)	35.7 (28.8–44.4)	36.2 (29.5–44.4)
East Africa							
Kenya	6 April 2020	88.89	18 915	8117 (62.2)	3672 (62.6)	35.2 (28.6–44.2)	35.4 (28.6–44.2)
Uganda	30 March 2020	93.52	11 638	5216 (60.6)	1926 (63.7)	30.5 (25.0–38.0)	30.7 (25.5–38.9)
North America							
Canada	1 April 2020	76.39	348	42 (17.5)	19 (17.6)	37.6 (29.9–46.8)	35.6 (30.1–44.9)
USA	21 March 2020	72.96	2823	463 (20.9)	143 (23.7)	35.7 (28.2–48.8)	34.6 (28.0–47.0)
Southern Africa							
Lesotho	29 March 2020	90.74	923	450 (61.3)	108 (57.1)	35.7 (28.5–43.6)	34.4 (28.0–45.9)
Mozambique	30 June 2020	80.56	9194	3445 (60.9)	2177 (61.6)	31.6 (25.7–40.6)	31.7 (25.6–41.0)
Malawi	18 April 2020	60.19	10 624	4841 (60.0)	1446 (56.7)	32.7 (26.3–39.5)	33.3 (26.9–39.8)
South Africa	26 March 2020	87.96	54 137	26 674 (63.4)	7524 (62.3)	33.4 (27.3–41.0)	33.6 (27.3–41.1)
Zambia	2 May 2020	70.83	43 563	18 711 (59.0)	7436 (62.8)	33.1 (27.2–40.1)	33.0 (27.1–40.1)
Zimbabwe	30 March 2020	87.96	3283	1546 (63.2)	534 (63.9)	34.8 (27.4–41.3)	34.7 (27.1–41.9)
West Africa							
Benin	30 March 2020	70.83	482	228 (62.3)	66 (56.9)	38.8 (32.2–47.4)	40.5 (32.2–53.0)
Burkina Faso	27 March 2020	89.81	706	308 (68.4)	164 (64.1)	40.4 (33.5–49.1)	39.2 (31.9–47.3)
Côte d’Ivoire	24 March 2020	80.56	1416	680 (67.3)	245 (60.3)	39.6 (32.8–48.0)	38.9 (30.8–46.2)
Nigeria	30 March 2020	85.65	1242	306 (57.3)	448 (63.3)	38.9 (30.0–45.5)	51.0 (46.1–56.8)
Togo	2 April 2020	73.15	1909	(58.0)	305 (27.4)	38.0 (30.0–45.0)	30.4 (24.5–40.0)

* Initiated between 2 years before and 1 year after start of pandemic for each country.

† Pre-COVID-19 period in each country defined as period between date of peak Stringency Index and 2 years before this date.

ART, antiretroviral therapy.

### Demographics of PLWH included in the analysis

The sex at birth and the median age of included PLWH who initiated ART prior to and during the pandemic are presented by country in table 1. In most countries, the proportion of women who initiated ART remained relatively stable during the pandemic. The median age of PLWH who initiated ART was comparable prior to and during the pandemic.

### Number of ART initiations and the proportion of timely initiations prior to and during the pandemic

The number of ART initiations and the proportion of timely initiations before and during the pandemic period are presented in tables2  3, respectively. Throughout the study period, the number of initiations per country ranged from 119 (Korea) to 54 137 (South Africa) and the proportion of timely initiations varied from 30.2% (Benin) to 99.2% (Mozambique).

**Table 2:** Number of observations by period and estimated changes in number of ART initiations per week during COVID-19 pandemic period, by region/country

	Number of ART initiations in the pre-COVID-19 period N	Number of ART initiations in the pandemic period N	ART initiations	
			Estimated changes in number of ART (p) per week with 95% CIs	P value
Asia/Pacific				
Hong Kong	272	84	−1.2 (−4.2 to 1.8)	0.45
Indonesia	403	153	−0.8 (−4.8 to 3.3)	0.71
India	939	186	**−5.0 (−9.2 to −0.7**)	**0.02**
Japan	164	114	−0.9 (−2.6 to 0.8)	0.29
Cambodia	296	103	3.1 (−4.3 to 10.5)	0.41
Korea	75	44	−0.5 (−2.7 to 1.7)	0.66
Malaysia	160	81	−2.6 (−7.3 to 2.0)	0.27
Philippines	206	12	3.9 (−3.8 to 11.6)	0.32
Thailand	789	299	1.5 (−2.7 to 5.8)	0.48
Central Africa				
Burundi	807	349	1.5 (−3.4 to 6.4)	0.56
Cameroon	1605	693	2.4 (−3.7 to 8.6)	0.44
Democratic Republic of Congo	797	502	−0.2 (−5.2 to 4.8)	0.93
Rwanda	1884	765	**−10.0 (−18.6 to −1.4**)	**0.02**
Latin America				
Brazil	458	171	0.7 (−1.2 to 2.6)	0.47
Chile	713	180	0.6 (−3.0 to 4.3)	0.74
Haiti	2208	646	−1.3 (−6.4 to 3.8)	0.61
East Africa				
Kenya	13 047	5868	−8.8 (−79.9 to 62.3)	0.81
Uganda	8613	3025	−20.9 (−58.8 to 16.9)	0.28
North America				
Canada	240	108	−0.8 (−2.2 to 0.5)	0.23
USA	2219	604	−2.7 (−15.1 to 9.7)	0.67
Southern Africa				
Lesotho	734	189	−3.3 (−8.0 to 1.3)	0.16
Mozambique	5661	3533	2.1 (−12.6 to 16.9)	0.78
Malawi	8072	2552	**−33.4 (−54.1 to −12.3**)	**0.002**
South Africa	42 055	12 082	**−130.8 (−188.6 to −73.1**)	**<0.001**
Zambia	31 724	11 839	11.5 (−40.4 to 63.3)	0.67
Zimbabwe	2447	836	**−12.9 (−20.0 to −5.8**)	**<0.001**
West Africa				
Benin	366	116	−0.4 (−2.9 to 2.1)	0.74
Burkina Faso	450	256	3.6 (−4.0 to 11.1)	0.35
Côte d’Ivoire	1010	406	−2.3 (8.4 to 3.7)	0.46
Nigeria	534	708	−9.4 (−20.1 to 1.3)	0.08
Togo	795	1114	**−19.6 (−39.1 to −0.1**)	**0.05**

Note: Statistically significant estimates are shown in bold (p < 0.05).

ART, antiretroviral therapy.

**Table 3:** Proportions of PLWH timely initiated by period and estimated changes in proportions of timely ART initiations per week during COVID-19 pandemic period, by region/country

	Proportion of timely initiated pre-COVID-19 period %	Proportion of timely initiated during pandemic period %	Proportions	
			Estimated changes in proportions of timely ART initiations (pp) per week with 95% CIs	P value
Asia/Pacific				
Hong Kong	32.0	41.0	−10.2 (−63.1 to 42.8)	0.71
Indonesia	34.5	45.1	8.1 (−48.7 to 65.0)	0.78
India	61.4	66.6	−7.4 (−92.5 to 87.5)	0.88
Japan				
Cambodia	42.6	89.5	−12.9 (−71.5 to 45.6)	0.67
Korea				
Malaysia				
Philippines				
Thailand	47.6	59.4	1.3 (−23.6 to 26.1)	0.92
Central Africa				
Burundi	88.1	92.4	12.2 (−8.2 to 32.5)	0.24
Cameroon	96.1	98.0	3.8 (−1.5 to 9.0)	0.16
Democratic Republic of Congo	89.2	88.1	−0.3 (−20.8 to 20.2)	0.98
Rwanda	81.6	91.2	−1.2 (−17.12 to 14.8)	0.89
Latin America				
Brazil			–	–
Chile	94.0	92.5	9.2 (−3.3 to 21.6)	0.15
Haiti	98.0	99.2	3.3 (−2.6 to 9.2)	0.28
East Africa				
Kenya	87.3	95.0	**4.2 (0.3 to 8.1)**	**0.04**
Uganda	87.0	90.2	9.0 (−1.5 to 19.5)	0.09
North America				
Canada	41.4	54.2	19.8 (−55.6 to 95.1)	0.61
USA	60.3	66.9	14.1 (−17.9 to 46.1)	0.39
Southern Africa				
Lesotho	98.7	99.3	−0.3 (−1.3 to 0.7)	0.53
Mozambique	99.0	99.6	**2.7 (0.5 to 4.9)**	**0.02**
Malawi	96.3	96.5	−3.6 (−7.5 to 0.3)	0.07
South Africa	90.3	93.8	−0.5 (−2.8 to 1.9)	0.68
Zambia	93.0	96.0	0.01 (−2.9 to 2.9)	0.99
Zimbabwe	96.5	98.4	−1.1 (−7.4 to 5.2)	0.73
West Africa				
Benin	22.6	45.9	21.9 (−14.0 to 57.9)	0.23
Burkina Faso	59.3	97.2	−7.0 (−18.8 to 4.8)	0.25
Côte d’Ivoire	91.7	89.3	−2.9 (−18.7 to 13.0)	0.72
Nigeria	43.9	26.7	1.5 (−85.7 to 88.7)	0.97
Togo	70.1	99.1	−8.4 (−19.0 to 2.1)	0.12

Note: Statistically significant estimates are shown in bold (p < 0.05).

ART, antiretroviral therapy; PLWH, people living with HIV.

### Changes in number of ART Initiations before and after the onset of the pandemic

**Figure 2:**
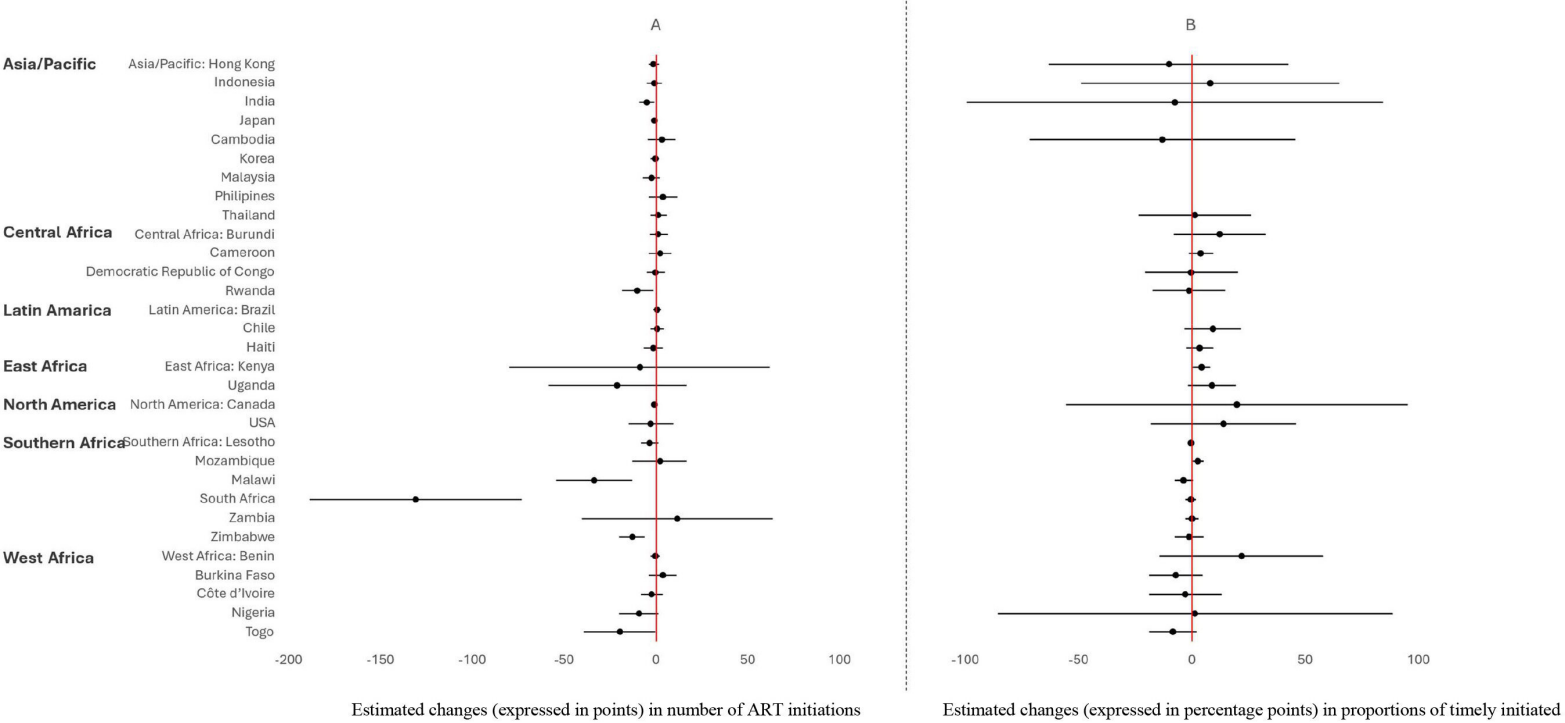
Estimated changes (and 95% CI) in number of ART initiations (**A**) proportions of timely initiated (**B**), during COVID-19 pandemic period, by region/country (countries with an insufficient number of observations were not included due to a lack of power). ART, antiretroviral therapy.

An overview of changes in number of ART initiations per week and in proportion of timely initiations by country/region before and after the onset of the pandemic is presented in figure 2A,B and tables 2 and 3. The number of ART initiations remained stable in 25 of the 31 countries included in the analysis. However, six countries experienced significant decreases in the number of ART initiations during the pandemic period: India (−5.0 points (p), 95% CI −9.2 to −0.7), Rwanda (−10.0 p, 95% CI −18.6 to −1.4), Malawi (−33.4 p, 95% CI −54.1 to −12.3), South Africa (−130.8 p, 95% CI −188.6 to −73.1), Zimbabwe (−12.9 p, 95% CI −20.0 to −5.8) and Togo (−19.6 p, 95% CI −39.1 to −0.1).

The proportion of timely initiations remained stable in 24 of the 26 countries included in the analysis. Notably, small improvements in the proportion of timely ART initiations during the pandemic were observed in Kenya and Mozambique. In Kenya, the proportion increased by 4.2 pp (95% CI +0.3 to +8.1), while in Mozambique, it rose by 2.7 pp (95% CI +0.5 to +4.9).

## Discussion

In most countries, we did not find the number of ART initiations in those newly enrolled nor their timeliness to be significantly different in the 52 weeks after the pandemic onset compared with the 104 weeks prior. This is reassuring as it suggests that clinics were (on average over the year after pandemic onset) able to maintain access to ART for newly diagnosed PLWH and uphold a ‘Test and Treat’ strategy, despite generally stringent responses to the COVID-19 pandemic. We attribute this stability to the resilience of clinics in the face of pandemic-related challenges and hypothesise that the prioritisation of non-COVID-19 services, including HIV services, the rapid implementation of adaptive strategies—such as streamlined ART initiation and reduced counselling requirements—contributed to maintaining timely access to treatment. However, six countries (India, Rwanda, Malawi, South Africa, Zimbabwe and Togo) experienced significant declines in the number of ART initiations in the year after pandemic onset. As many are home to a significant number of PLWH, changes in absolute numbers of ART initiations would be expected to be larger and more obvious. Further research is required to better understand whether observed declines in ART initiation were due to the severity of public health and social measures such as mobility restrictions, the dual burden on health services of HIV and COVID-19 infections, the reallocation of resources, underlying health system weaknesses or a combination of these factors.

In settings where similar analyses have been conducted (eg, the United States, Asia Pacific region, Kenya), our findings regarding the overall stability of ART initiations and/or their timeliness appear to confirm published reports.^29^,^31^ Our results may be evidence of health system and service adaptability, that is, the ability to respond to changing needs and challenges during the first year of the COVID-19 pandemic. In its report, the Global Fund noted that strong and adaptive health systems played a crucial role in maintaining ART services during the COVID-19 pandemic.^32^ Previous research conducted within the IeDEA consortium also supports this explanation. Although most local IeDEA investigators/healthcare providers in these regions reported being subject to pandemic-related restrictions and clinic operations being hindered (eg, reconfiguration of hospital/clinic space, reduced provider availability because of illness, self-isolation, quarantine or because of reassignment related to the pandemic), few, at least in low-income settings, reported suspending routine ART clinics or ART initiation for new patients.^33^ In Latin America specifically, the majority of investigators/healthcare providers (11 of 13) indicated they too had maintained ART initiation services.^34^ Furthermore, many clinics adopted strategies to mitigate the pandemic’s impact on adherence, including multimonth ART dispensing and community-based ART delivery.^35 36^ In 30% of facilities, same-day ART initiation was expanded, reducing the risk of loss to follow-up, which was particularly critical during the pandemic.^33 37^ These adaptations, widely promoted under Differentiated Service Delivery Models, may have been instrumental, allowing providers to prioritise ART initiation for newly diagnosed individuals during the COVID-19 pandemic. While implemented at a time when clinics were stressed by COVID-19, these adaptations could streamline services and improve long-term efficiency beyond the crisis.^38 39^ This interpretation was also put forth in a previous report of the pandemic’s impact on clinics supported by the US Centers for Disease Control and Prevention through the President’s Emergency Plan for AIDS Relief in which adaptive service delivery models, technology, and virtual platforms for client engagement and site-level monitoring facilitated a rapid recovery from initial declines in ART initiations between March and June 2020. Our findings, together with country-specific studies conducted during the pandemic, support the integration and scale-up of differentiated and flexible service delivery models in HIV care programmes. In South Africa and Zimbabwe, countries with large and well-established HIV treatment programmes serving approximately 7.6 and 1.3 million PLWH, respectively.^40^ ART initiations declined by 130.8 points per week (−188.6 to −73.1) in South Africa and −12.9 points (−20.0 to −5.8) in Zimbabwe in the first year of the pandemic compared with pre-pandemic levels. This suggests that even countries with mature programmes were not immune to pandemic-related disruptions. These findings align with Dorward *et al*, which found both HIV testing and ART initiations to have decreased significantly following the national lockdown, with a gradual recovery over the following months in KwaZulu-Natal, South Africa.^19^ Not only were public health and social measures strict in South Africa (Oxford Stringency Index Score of 87.96), including severe mobility restrictions which limited access to clinics, according to Dorward *et al*, healthcare workers were also reassigned to support pandemic response.^19^ In Zimbabwe, where over 90% of PLWH were on ART prior to the pandemic, we also observed a decrease in ART initiations. A nationwide lockdown was imposed on 30 March 2020, restricting movement and suspending public transportation, making clinic access more difficult. Additional restrictions were introduced in January and June 2021, prolonging service interruptions. Furthermore, healthcare worker strikes—first a doctors’ strike pre-pandemic (September 2019–January 2020), followed by a nurses’ strike (June–September 2020) partly due to personal protective equipment shortages—may have contributed to delays in HIV diagnosis and ART initiation. The combination of testing disruptions, healthcare workforce shortages and mobility restrictions, occurring in the first year of the pandemic, likely contributed to the decline in ART initiations observed.^41 42^ In India, home to 2.3 million PLWH, a similar pattern was observed. Public and health and social measures were among the most stringent registered globally (Oxford Stringency Index Score of 100). There is evidence that the mobility restrictions hampered access to clinics, resulting in disruptions to routine health services.^43^ Ray *et al* have reported that HIV clinics were understaffed or temporarily closed, offering an explanation of the observed declines in ART initiations. Finally, in Rwanda, another highly successful programme, although in a setting with a lower burden of disease compared with Southern and Eastern Africa, strict measures were enacted (Oxford Stringency Index Score=90), disrupting programmes targeting key populations and possibly contributing to the decline in ART initiation observed here.^42 44 45^ Our findings underscore the need for policy frameworks that safeguard HIV service continuity during systemic shocks, including pandemics, conflicts or climate-related crises.

We also observed declines in ART initiations in countries where public health and social measures were not as strict. In Malawi, the Oxford Stringency Index Score peak was 60.19 and yet we found ART initiation to have decreased by 33.4 pp (−54.1 to −12.3), suggesting that factors beyond the severity of measures affected service accessibility and availability. One potential explanation is that pre-existing health system constraints—such as workforce shortages, inadequate infrastructure and dependence on external support—may have left HIV-related services less able to absorb the additional strain imposed by the COVID-19 pandemic period. Evidence suggests that Malawi’s healthcare system faced both challenges associated with availability (limited staffing, supply chain disruptions, clinic closures) and accessibility (restricted mobility) during the pandemic. There is also evidence of a drop in HIV testing and inconsistent recovery, possibly due to pre-existing constraints,^46^,^48^ resulting in fewer new patients presenting for care. These findings highlight that the resilience of HIV programmes depends not only on the severity of measures but also on the underlying strength and preparedness of the health system. Policymakers should prioritise long-term investments in workforce development, infrastructure and supply chains, as well as strategies to enhance local ownership of HIV services.

In Togo, ART initiations decreased by −19.6 pp (−39.1 to −0.1) compared with pre-pandemic levels, with declines being attributed to similar availability and accessibility challenges faced by Malawi. A recent study of the pandemic’s impact on health services for HIV, TB and Malaria in Togo, conducted using interrupted time series analyses, found a significant downward trend in number of ART initiation during the pandemic (IRR 0.91, 95% CI 0.89 to 0.93, p<0.001) but, unlike us, it did not report a significant decrease overall. This discrepancy may stem from methodological differences. While Konu *et al*’s study assessed gradual monthly trends, our approach assessed ART initiations after the pandemic onset compared with 2 years before using an RDD, thus focusing on immediate changes at a given threshold (pandemic onset). This distinction may explain why we have detected a significant reduction in ART initiations, while their analysis identified a downward trend without clear discontinuity.^49^

While previous research from sub-Saharan Africa has shown that countries with higher Oxford Stringency Index scores often faced declines in HIV testing and new ART initiations, our findings reveal a more nuanced picture.^50 51^ The stability observed in some high-stringency settings raises questions about the actual implementation and enforcement of public health and social measures and their true impact on the continuity of care. The Oxford Stringency Index provides a standardised measure of national policies but does not account for the actual implementation or enforcement of measures at the local level. In practice, the degree to which these restrictions were applied varied across and within countries, influenced by governance, infrastructure and public compliance. Moreover, the nature of the measures themselves likely mattered—restrictions that directly affected individuals’ mobility and thus their access to clinics may have had a greater impact on ART initiation than general measures such as limits on gatherings or mandates related to masking or hygiene.

Furthermore, the Oxford Stringency Index also does not capture health system adaptability, such as local service delivery adjustments to maintain ART access despite restrictions. The stability observed in some high-stringency settings suggests that pre-existing infrastructure, rapid response capacity and service delivery innovations may have played an equal, if not more important, role in sustaining ART initiations than policy strictness alone. Exploring these dynamics could provide valuable insights into which service delivery adaptations helped maintain ART access and how. A nuanced understanding of said dynamics would inform broader discussions on health system strengthening and resilience generally and as they pertain to HIV services.^52^

### Strengths and limitations

This study’s main strength lies in its geographic scope. By including data from 31 countries from different regions, it provides insight into how ART initiation was affected in both high and low resource settings at the onset of the COVID-19 pandemic. However, clinics participating in the IeDEA consortium may not be representative of the standard of HIV care in countries, potentially limiting the generalisability of our findings.

We used the Oxford Stringency Index to define the onset of the pandemic. This method not only allowed for a standardised means of accounting for the severity of the main public health and social measures in each setting but also enabled investigation of the underlying hypothesis: that public health response may have indirectly affected the delivery of other (non-COVID-19 related) services including those for people presenting at clinics with HIV. However, we acknowledge that using the date corresponding to the peak Oxford Stringency Index Score, occurring between 1 January 2020 and 30 June 2020, as a proxy for pandemic onset may not perfectly reflect the true timing of restrictions in all countries—particularly in settings where stringency peaked after public health and social measures were already in place or where enforcement varied locally.

Furthermore, our analysis focused on changes in ART initiations before and during the pandemic but did not account for pre-pandemic ART initiation rates in each country. In mature programmes, sustaining high levels of ART initiations during a global health crisis may have been more complex, particularly where service delivery was already operating at capacity or where programme priorities had already shifted toward retention and re-engagement rather than new initiations. This limitation highlights the importance of considering historical trends and baseline differences when interpreting the impact of the pandemic. Notably, countries with lower weekly ART initiation counts may have had reduced statistical power to detect significant changes, potentially limiting the precision of effect estimates. And finally, our study does not account for the pandemic’s longer-term effects, nor does it capture other critical steps in the cascade of care (eg, case finding and viral load monitoring).^53^ It also does not reflect broader programme shifts that occurred during this period, such as efforts to re-engage individuals previously disengaged from care or prioritise retention in care rather than new initiations—as was the case in countries like South Africa, where the proportion of people never started on ART had become relatively small. These important strategies are not captured in our outcome, focused solely on new ART initiations.

### Conclusions

Despite stringent public health and social measures, ART initiations remained stable in most settings during the first year of the COVID-19 pandemic, highlighting both the resilience and the adaptability of numerous HIV programmes/services globally. Nevertheless, the significant decline in absolute numbers of ART initiations in some settings, home to millions of PLWH, raises concerns. A deeper understanding of the factors that contributed to sustaining ART initiations, particularly in settings with stringent public health and social measures, is needed. We must then translate these lessons into preparedness strategies, resource allocation priorities and policies that safeguard HIV service continuity in future health emergencies, in line with WHO recommendations.

## Supplementary material

10.1136/bmjopen-2025-112903 online supplemental file 1

## Data Availability

Data are available on reasonable request.
