# Supplementary material for: Effect of COVID-19 pandemic on ART access and timely initiation in people living with HIV in 31 countries: a regression discontinuity design study
Source: BMJ Open. 2026 Mar 10;16(3):e112903. doi: 10.1136/bmjopen-2025-112903 (PMC12983828; doi:10.1136/bmjopen-2025-112903)

# Annex

### Annex 1 : Definition of the pandemic onset based on the Stringency Index

|  | Start of pandemic period based on **national** first PHSM  (A) | Date of highest stringency score in the **first 6 months of 2020**  (B) | Peak Stringency score in the **first 6 months of 2020**  (B) | Date of highest stringency score from **January 2020 and June 2021**  (C) | Peak Stringency score from **January 2020 and June 2021**  (C) |
| --- | --- | --- | --- | --- | --- |
| **Asia/Pacific** |  |  |  |  |  |
| Hong Kong | 25/03/2020 | 06/04/2020 | 66.67 | 04/12/2020 | 71.30 |
| Indonesia | 17/03/2020 | 24/04/2020 | 80.09 | 24/04/2020 | 80.09 |
| India | 24/03/2020 | 22/03/2020 | 100 | 22/03/2020 | 100 |
| Japan | 26/03/2020 | 16/04/2020 | 47.22 | 07/06/2021 | 55.09 |
| Cambodia | 27/01/2020 | 10/04/2020 | 76.85 | 15/04/2021 | 83.8 |
| Korea | 29/05/2020 | 06/04/2020 | 82.41 | 06/04/2020 | 82.41 |
| Malaysia | 11/05/2020 | 27/03/2020 | 78.70 | 30/06/2021 | 91.67 |
| Philippines | 24/03/2020 | 22/03/2020 | 100 | 22/03/2020 | 100 |
| Thailand | 26/03/2020 | 03/04/2020 | 76.85 | 03/04/2020 | 76.85 |
| **Central Africa** |  |  |  |  |  |
| Burundi | 09/07/2020 | 22/03/2020 | 13.89 | 16/02/2021 | 27.31 |
| Cameroon | 02/04/2020 | 18/04/2020 | 71.3 | 18/04/2020 | 71.3 |
| Democratic Republic of Congo | 24/03/2020 | 06/04/2020 | 80.56 | 06/04/2020 | 80.56 |
| Rwanda | 08/03/2020 | 21/03/2020 | 90.74 | 21/03/2020 | 90.74 |
| **Latin America** |  |  |  |  |  |
| Brazil | 13/03/2020 | 05/05/2020 | 81.02 | 05/05/2020 | 81.02 |
| Chile | 26/03/2020 | 15/05/2020 | 81.02 | 03/07/2020 | 90.28 |
| Haiti | 25/03/2020 | 19/04/2020 | 93.52 | 19/04/2020 | 93.52 |
| **East Africa** |  |  |  |  |  |
| Kenya | 06/04/2020 | 06/04/2020 | 88.89 | 06/04/2020 | 88.89 |
| Uganda | 27/04/2020 | 30/03/2020 | 93.52 | 30/03/2020 | 93.52 |
| **North America** |  |  |  |  |  |
| Canada | 23/03/2020 | 01/04/2020 | 76.39 | 01/04/2020 | 76.39 |
| USA | 16/03/2020 | 21/03/2020 | 72.96 | 16/11/2020 | 75.46 |
| **Southern Africa** |  |  |  |  |  |
| Lesotho | 20/07/2020 | 29/03/2020 | 90.74 | 29/03/2020 | 90.74 |
| Mozambique | 14/03/2020 | 30/06/2020 | 80.56 | 30/06/2020 | 80.56 |
| Malawi | 09/04/2020 | 18/04/2020 | 60.19 | 08/08/2020 | 64.81 |
| South Africa | 19/04/2020 | 26/03/2020 | 87.96 | 26/03/2020 | 87.96 |
| Zambia | 14/04/2020 | 02/05/2020 | 70.83 | 02/05/2020 | 70.83 |
| Zimbabwe | 24/03/2020 | 30/03/2020 | 87.96 | 30/03/2020 | 87.96 |
| **West Africa** |  |  |  |  |  |
| Benin | 23/03/2020 | 30/03/2020 | 70.83 | 30/03/2020 | 70.83 |
| Burkina Faso | 27/03/2020 | 27/03/2020 | 89.81 | 27/03/2020 | 89.81 |
| Côte d’Ivoire | 09/04/2020 | 24/03/2020 | 80.56 | 24/03/2020 | 80.56 |
| Nigeria | 30/03/2020 | 30/03/2020 | 85.65 | 30/03/2020 | 85.65 |
| Togo | 21/03/2020 | 02/04/2020 | 73.15 | 02/04/2020 | 73.15 |


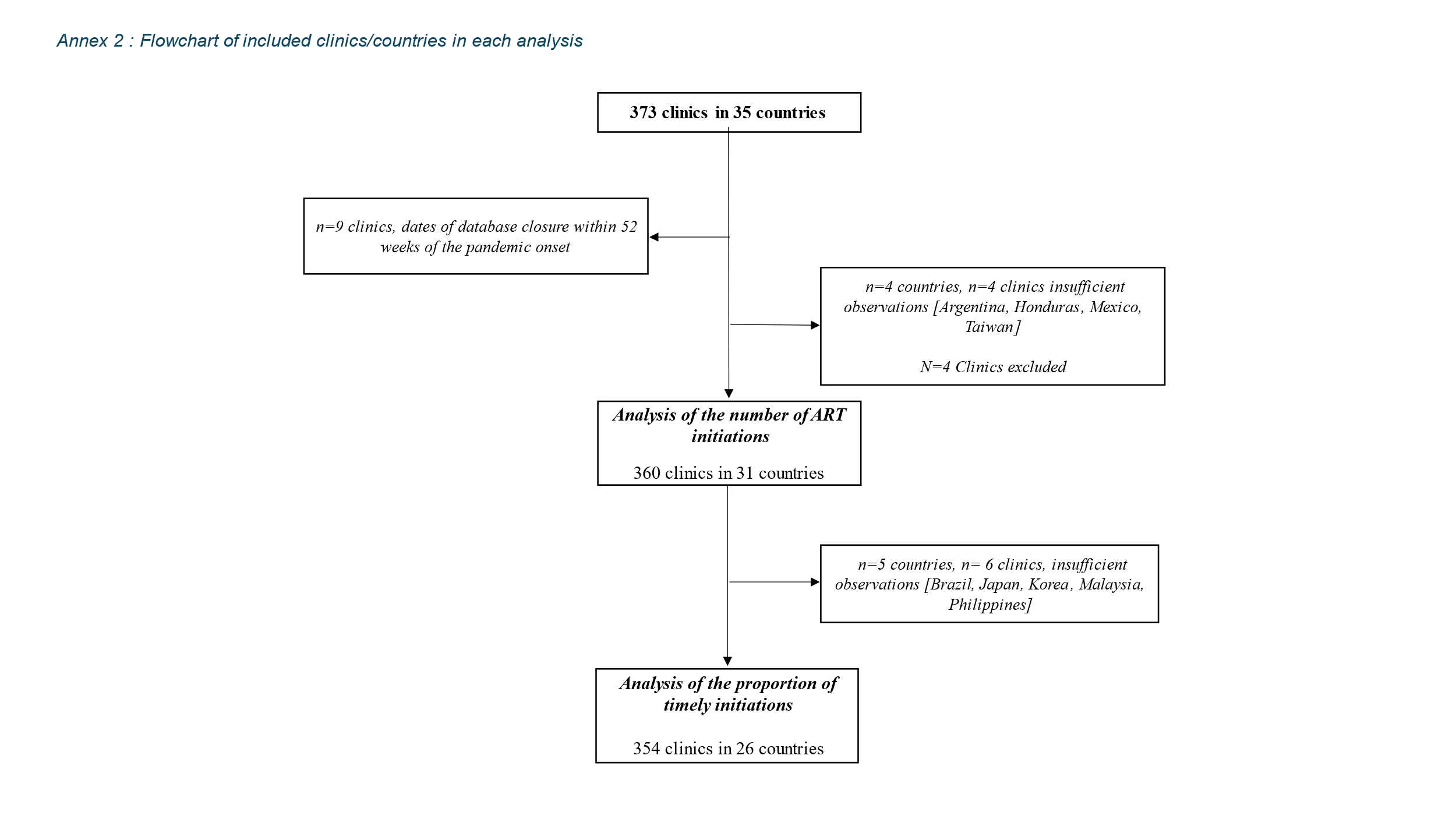

Supplement: online supplemental file 1 [file bmjopen-16-3-s001.docx]
