# Supplementary material for: Effect of COVID-19 pandemic on ART access and timely initiation in people living with HIV in 31 countries: a regression discontinuity design study
Source: BMJ Open. 2026 Mar 10;16(3):e112903. doi: 10.1136/bmjopen-2025-112903 (PMC12983828; doi:10.1136/bmjopen-2025-112903)
Supplement: Supplementary file 2 [file bmjopen-2025-112903.reviewer_comments.pdf]

PEER REVIEW HISTORY

BMJ Open publishes all reviews undertaken for accepted manuscripts. Reviewers are asked to complete a checklist review form and are provided with free text boxes to elaborate on their assessment. These free text comments are reproduced below.

This paper was submitted to another journal from BMJ but declined for publication following peer review. The authors addressed the reviewers' comments and submitted the revised paper to BMJ Open. The paper was subsequently accepted for publication at BMJ Open.

ARTICLE DETAILS

Title (Provisional)

Effect of COVID-19 Pandemic on ART Access and Timely Initiation in People living with HIV in 31 countries : A Regression Discontinuity Design Study

Authors

Ben Farhat, Jihane; Messou, Eugène; Borse, Rohidas; Varela Bustillo, Diana; Madimabe, Metsekae; Nash, Denis; Byakwaga, Helen; Shah, N. Sarita; Ezechi , Oliver; Pujari, Sanjay; Veloso, Valdiléa G.; Hobbins, Michael; Murenzi, Gad; Mkwashapi, Denna; Hogan, Brenna; Choi, Jun Yong; Minga, Albert; Crabtree-Ramírez, Brenda; Twizere, Christella; Diero, Lameck; Haw, Jason; Bolton-Moore, Carolyn; Cortes, Claudia P.; Lee, Man-Po; Mbewe, Safari; Brazier, Ellen; Humphrey, John M; Althoff, KN; Bonnet, Fabrice; Barger, Diana; Jaquet, Antoine; International epidemiology Databases to Evaluate AIDS, IeDEA

VERSION 1 - REVIEW

**Reviewer**	**1**
**Name**	**Reviewer 1**
**Affiliation**	
**Date**	**08-Oct-2025**
**COI**	**No competing interest**

Comments not published pending reviewer's consent

**Reviewer**	**2**
**Name**	**Reviewer 2**
**Affiliation**	
**Date**	**08-Oct-2025**

COI **No competing interest**

Comments not published pending reviewer’s consent

Reviewer **3**

Name **Reviewer 3**

Affiliation

Date **08-Oct-2025**

COI **No competing interest**

Comments not published pending reviewer’s consent

Reviewer **4**

Name **Reviewer 4**

Affiliation

Date **08-Oct-2025**

COI **No competing interest**

Comments not published pending reviewer’s consent

VERSION 2 - REVIEW

Reviewer **5**

Name **Kazibwe, Andrew**

Affiliation **Makerere University, Medicine**

Date **09-Dec-2025**

COI **None**

I thank the authors for documenting the impact of COVID-19 containment measures on anti-retroviral therapy initiation and timeliness. Please find below some observations:

(a) The study objective/research gap is not clear. What was the purpose of conducting this study? I advise the authors to harmonise the content of the last two paragraphs of the background section. In one paragraph, the authors describe a limited evidence of the influence of "stringency" of COVID-19 containment measures on ART treatment initiation; while in another, they describe a need to further elucidate the epidemiology of HIV

treatment during the COVID-19 pandemic. While there are overlaps in these phenomena, they are not entirely synonymous.

(b) In the abstract - conclusion - the authors highlight the need to study "settings with stringent public health and social measures" yet there was no attempt to grade the stringency of settings, nor study relationships between stringency and ART initiation nor timeliness. This is erroneous.

(c) The authors used the Oxford Stringency Index as a proxy to determine the onset of the COVID-19 pandemic in the different settings. There are limitations to this, that ought to be acknowledged. First, peak of stringency does not perfectly reflect pandemic onset, neither was it heterogenous across settings. Authors need to explain how they dealt with settings where first public health and social measures seem to have commenced after the peak stringency date, such as Korea and Malaysia, as shown in annex 1.

(d) I encourage the authors to provide references for key assertions in the background section, such as the limitation of observational studies.

(e) In the background and methodology sections, the authors acknowledge limitations of retrospective observational studies, that, to me, are not addressed in the present study. What, then, was the research gap that the authors sought to address?

(f) How did the authors define the date of presentation to the clinic? Was this the date of HIV diagnosis?

(g) I suggest that the authors exclude recommendations on further research from the first paragraph of the discussion section. The first paragraph of the discussion section should restate the study aim and summarise the key study findings. Please paraphrase paragraphs in the discussion section to present a concise message per paragraph in the discussion section.

**Reviewer**	**6**
**Name**	**Bischoff, Jenny**
**Affiliation**	**University of Bonn, Internal Medicine**
**Date**	**21-Dec-2025**
**COI**	**None**

Farhat et al. describe the impact of the COVID pandemic on HIV testing and timely treatment initiation in 31 countries. The authors have extensively revised the manuscript. In my opinion, the current version can be accepted as is.

VERSION 2 - AUTHOR RESPONSE

Editor (E) Reviewer (R)	Comments	Reply
R1.1	The study objective/research gap is not clear. What was the purpose of conducting this study? I advise the authors to harmonise the content of the last two paragraphs of the background section.<br><br>In one paragraph, the authors describe a limited evidence of the influence of "stringency" of COVID-19 containment measures on ART treatment initiation; while in another, they describe a need to further elucidate the epidemiology of HIV treatment during the COVID-19 pandemic. While there are overlaps in these phenomena, they are not entirely synonymous.	We thank the reviewer for this thoughtful comment. While we felt the study objective was already clearly conveyed in the last paragraph of the introduction, we have rephrased it slightly to enhance clarity and ensure better alignment across the background section. We hope this revision improves the overall readability.<br><br>*Page 6 : "As the Test and Treat strategy relies not only on the availability of health system resources such as diagnostics, ART, and trained personnel (25)· but also on their accessibility, including the ability of individuals to physically reach care, we hypothesized that the unprecedented circumstances of the COVID-19 pandemic may have disrupted both availability and accessibility.* ***These disruptions could, in turn, have affected the delivery of non-COVID-19 services, including key aspects of HIV care such as ART initiations among those presenting for care and/or their timeliness.*** *To investigate this hypothesis, we apply a regression discontinuity design to assess the causal impact of the COVID-19 pandemic onset on the number and timeliness of ART initiations at clinics in 31 countries and* ***interpret these results in light of the severity of public health and social measures implemented in each setting."***
R1.2	In the abstract - conclusion - the authors highlight the need to study "settings with stringent public health and social measures" yet there was no attempt to grade the stringency of settings, nor study relationships	We thank the reviewer for this helpful observation. While our primary aim was not to model the association between stringency and ART outcomes directly, we used the **peak Oxford Stringency Index** as a

	between stringency and ART initiation nor timeliness. This is erroneous.	**standardized time anchor** to define the start of the pandemic in each country, thereby enabling cross-country comparisons in timing. However, we agree that the abstract's phrasing may have inadvertently suggested a causal analysis between stringency and ART outcomes. We have tried to be more transparent about our use of the Oxford Stringency Index in the analysis and the interpretation of the results.<br><br>We have removed the following statement from page 6 : *"Finally, to our knowledge, few studies have investigated how the stringency of public health and social measures influenced the delivery of healthcare in non–COVID-19 populations like those with HIV."*<br><br>We have also revised the conclusion in the abstract accordingly for greater accuracy and clarity.<br><br>Page 3 : "*A deeper understanding of the factors that contributed to sustaining ART initiations, particularly in settings with stringent public health and social measures, is needed. These insights should inform preparedness strategies, resource allocation, and policy development to ensure continuity of HIV services during future health emergencies, in line with World Health Organization recommendations.*<br><br>Indeed, despite the COVID-19 pandemic, some settings were able to maintain ART initiations, even where the public health and social measures were strict. We don't fully know which factors made this possible. Identifying these factors is important (learning what worked during the pandemic) and using those lessons to protect HIV services in the future.

R1.3	The authors used the Oxford Stringency Index as a proxy to determine the onset of the COVID-19 pandemic in the different settings. There are limitations to this, that ought to be acknowledged. First, peak of stringency does not perfectly reflect pandemic onset, neither was it heterogenous across settings. Authors need to explain how they dealt with settings where first public health and social measures seem to have commenced after the peak stringency date, such as Korea and Malaysia, as shown in annex 1.	We thank the reviewer for this helpful comment. We agree that using the peak Oxford Stringency Index score as a proxy for pandemic onset has limitations, particularly where policy responses and epidemiologic curves may not have aligned perfectly. We have acknowledged this in the *Strengths and limitations* section.<br><br>Page 16 : *"However, we acknowledge that using the date corresponding to the peak Oxford Stringency Index Score, occurring between January 1, 2020 and June 20, 2020, as a proxy for pandemic onset may not perfectly reflect the true timing of restrictions in all countries — particularly in settings where stringency peaked after public health and social measures were already in place or where enforcement varied locally."*
R1.4	I encourage the authors to provide references for key assertions in the background section, such as the limitation of observational studies.	Thank you for this helpful suggestion. We have added appropriate references to support statements regarding the limitations of observational studies, particularly those relying on year-over-year comparisons or descriptive temporal trends.
R1.5	In the background and methodology sections, the authors acknowledge limitations of retrospective observational studies, that, to me, are not addressed in the present study. What, then, was the research gap that the authors sought to address?	We thank the reviewer for this thoughtful comment. We respectfully clarify that the research gap is explicitly articulated and is addressed by the current study design.<br><br>The core gap we identified is that most early studies assessing COVID-19's impact on ART initiation relied on descriptive before–after comparisons or year-over-year summaries, which do not adequately control for underlying temporal trends and may lead to biased estimates. This is precisely the limitation to which we refer in the Background.

		Our study directly addresses this gap by:<br><br>- Using a Regression Discontinuity Design (RDD), a quasi-experimental approach recommended for improving causal inference in observational settings.<br><br>- Defining a clear intervention threshold (pandemic onset based on peak Stringency Index), allowing estimation of immediate changes in level and trend rather than simple descriptive comparisons.<br><br>- Pooling harmonised, individual-level data from 31 countries, overcoming the fragmentation of previous studies confined to single cities or programmes.
R1.6	How did the authors define the date of presentation to the clinic? Was this the date of HIV diagnosis?	We thank the reviewer for this question and welcome the opportunity to clarify. In our study, **the date of presentation to the clinic was defined as the date of enrolment in HIV care recorded in the IeDEA database**, which corresponds to the first documented clinic visit for HIV care within the participating site.
R1.7	I suggest that the authors exclude recommendations on further research from the first paragraph of the discussion section. The first paragraph of the discussion section should restate the study aim and summarise the key study findings. Please paraphrase paragraphs in the discussion section to present a concise message per paragraph in the discussion section.	Thank you for this thoughtful comment. We agree that the opening paragraph of the discussion should restate the aim and summarize the main findings. We respectfully note, however, that our first paragraph already follows this structure by (i) restating the purpose of the study, (ii) presenting the core findings, and (iii) briefly contextualizing these findings within the broader evidence base.

		The brief mention of areas where further research is needed appears at the end of this paragraph and serves to orient the reader toward the subsequent, more detailed interpretation. For these reasons, and to maintain coherence with the rest of the discussion, we believe that the current structure is appropriate and does not hinder clarity. We have therefore retained the paragraph as written.
R2.1	Farhat et al. describe the impact of the COVID pandemic on HIV testing and timely treatment initiation in 31 countries. The authors have extensively revised the manuscript. In my opinion, the current version can be accepted as is.	We thank the reviewer for their positive assessment of our revisions and for their supportive comment. We are pleased that the current version is considered acceptable, and we appreciate the reviewer's constructive feedback throughout the process.
